# Binding Interactions of Peptide Aptamers

**DOI:** 10.3390/molecules25246055

**Published:** 2020-12-21

**Authors:** Roger R. C. New, Tam T. T. Bui, Michal Bogus

**Affiliations:** 1 Vaxcine (UK) Limited, London Bioscience Innovation Centre, London NW1 0NH, UK; michalbogus@proximaconcepts.com; 2 Faculty of Science & Technology, Middlesex University, Hendon, London NW4 4BT, UK; 3 Centre for Biomolecular Spectroscopy and Institute of Pharmaceutical Science, King’s College, London SE1 1UL, UK; tam.bui@kcl.ac.uk

**Keywords:** peptide therapeutics, cyclic peptide, lipo-amino acid, hydrogen bond, fluorescence enhancement, peptide aptamer

## Abstract

Peptide aptamers are short amino acid chains that are capable of binding specifically to ligands in the same way as their much larger counterparts, antibodies. Ligands of therapeutic interest that can be targeted are other peptide chains or loops located on the surface of protein receptors (e.g., GCPR), which take part in cell-to-cell communications either directly or via the intermediary of hormones or signalling molecules. To confer on aptamers the same sort of conformational rigidity that characterises an antibody binding site, aptamers are often constructed in the form of cyclic peptides, on the assumption that this will encourage stronger binding interactions than would occur if the aptamers were simply linear chains. However, no formal studies have been conducted to confirm the hypothesis that linear peptides will engage in stronger binding interactions with cyclic peptides than with other linear peptides. In this study, the interaction of a model cyclic decamer with a series of linear peptide constructs was compared with that of a linear peptide with the same sequence, showing that the cyclic configuration does confer benefits by increasing the strength of binding.

## 1. Introduction

Peptides are recognised as a valuable class of potential therapeutic agents owing to their small size, complex structures, and compatibility with biological systems. Cyclic peptides are particularly regarded as an important subcategory within this group, since they have greater stability and less conformational ambiguity compared to linear peptides. In certain cases, this may confer advantages when binding to large proteins, and many groups have sought to compare the binding of linear and cyclic peptides to proteins [1,2,3,4]. The results have not been clear, probably because a degree of flexibility is often required for the peptide to adapt its configuration to the rigid protein structure. However, the rigidity of cyclic peptides may be beneficial when binding to linear peptides, a property that could be valuable, for example, in targeting loops on cell membrane proteins such as GPCR, or any stretch of linear peptide in a protein joining two regions of secondary structure. To the best of our knowledge, however, no studies have been formally conducted that demonstrate this important interaction of cyclic peptides with linear peptides. Such a demonstration is provided in this study.

**Figure 1:**
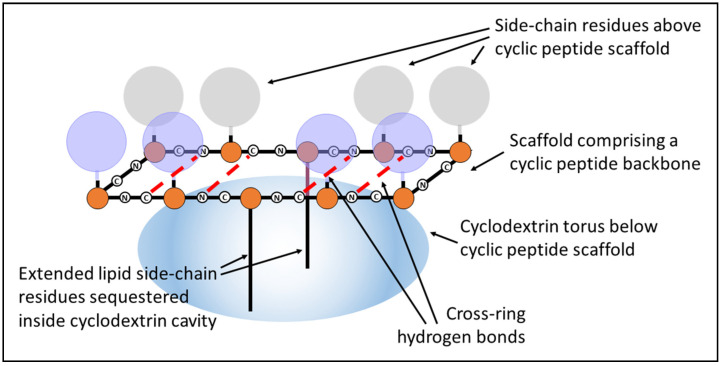
Structure and configuration of a peptide aptamer.

The cyclic peptides employed in this study are of the basic structure described as Lexicon peptides, as we outlined in previous papers [5,6]. These are usually decapeptides of the general structure shown in Figure 1, in which two amino acids with extended lipidic side chains are positioned on either side of the ring to introduce an internal constraint, which brings the two sides of the ring together as a result of noncovalent hydrophobic interactions. The extended lipidic side chains in these molecules are derived from cysteine lengthened by alkylation of the SH group with a C8 straight-chain hydrocarbon.

**Figure 2:**
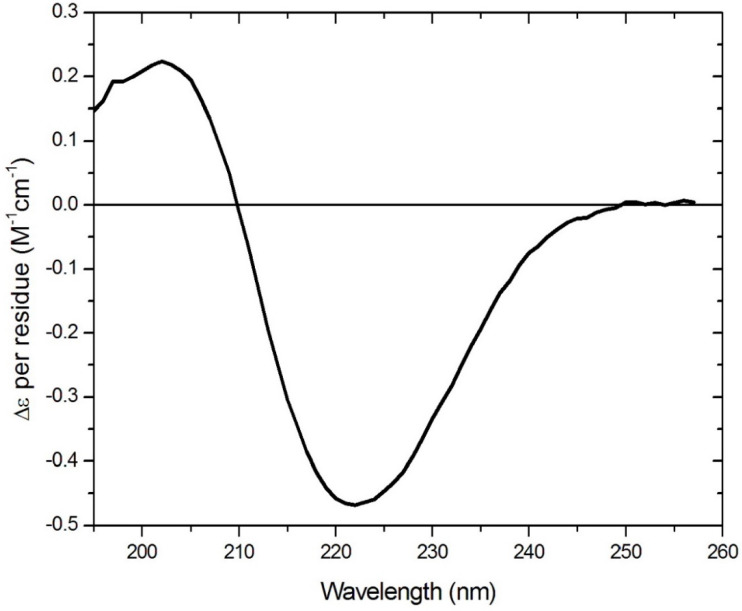
The far UV circular dichroism spectrum of a Lexicon cyclic peptide with the structure cyclo(sRC(C8)FsSrc(C8)fS), where L and D forms are represented by upper- and lower-case letters, respectively. The data are expressed as Molecular Circular Dichroism, or Δε per residue, using a mean molecular weight of 149. The single negative CD minimum at ~222 nm with the crossover at 210 nm is representative of the signature of a β-turn/β-turn structure [8,9].

This structure is stabilised by complexation with a cyclodextrin, which sequesters the lipid chains within the internal cavity of the polysaccharide torus and acts as a rigid support for the planar peptide ring. The rigidity of the aptamer is increased by cross-ring hydrogen bonding, resulting in a fixed-ladder configuration like that found in chains of an antiparallel beta-pleated sheet. Modelling (using both physical models and chemical computational software) has indicated no potential steric clash, although rotational freedom may be restricted slightly with large residues. Other than the circular dichroism (CD) data shown in Figure 2, molecular dynamics simulations provide support for the premise that the two long-chain hydrocarbons associate with each other to bring the opposite sides of the ring together closer than they would be in the absence of the chains. A CD spectrum (Figure 2) consistent with the presence in the ring of a beta turn is obtained for such peptides (confirmed using BestSel (http://bestsel.elte.hu/index.php)) [7], as would be required to allow the chain to turn sharply back on itself to achieve the cross-ring hydrogen bonding described above, and shown in Figure 1.

**Figure 3:**
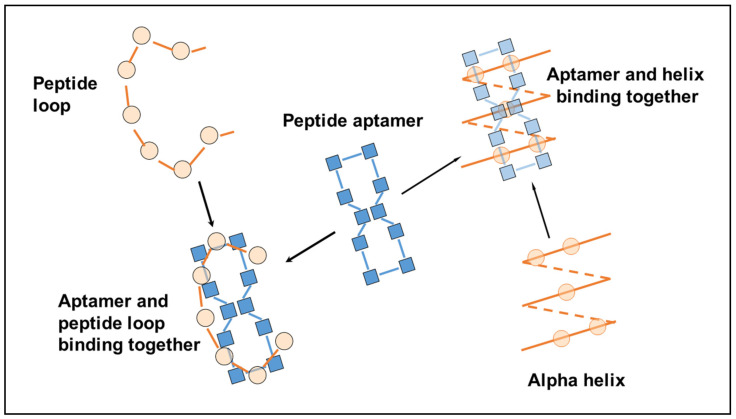
Peptide aptamers are a close fit with common structures in proteins.

The chirality of the amino acids is arranged so the lipid chains project downward from one side of the planar ring while the other amino acids face in the opposite direction and occupy the top surface, away from the cyclodextrin base. As such, all the amino acid side chains are available for binding with other molecules, and binding to peptides and proteins is particularly efficacious, since the spacing of the residues in the aptamer ring (i.e., the length of each peptide bond) matches the spacing of the residues in the target exactly, whether a loop, an alpha helix or a beta-pleated sheet, where the side chains can interleave with each other to form a close fit between the aptamer and the ligand (Figure 3).

**Figure 4:**
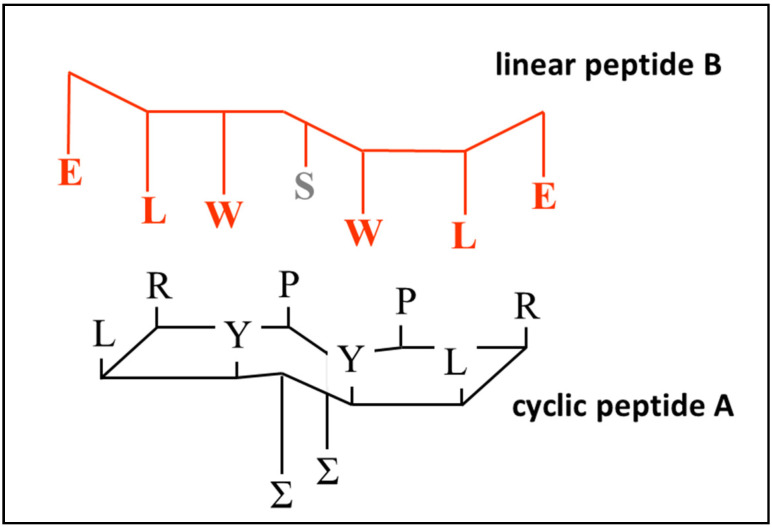
Sequence structure of the cyclic peptide aptamer (**A**) and linear peptide (**B**) employed in this study.

For the cyclic peptide to bind to a linear chain most effectively, interactions need to occur via inter-chain backbone hydrogen bonding, and between complementary side chain residues in the sequence. Therefore, although the most rigid conformation of the aptamer will be achieved when all four possible cross-ring hydrogens bonds are established, free hydrogen bonds available for bonding to a linear peptide can be created by including prolines in the aptamer ring, which will create free carbonyl groups that are no longer paired with N–H functionalities, making them available to bind to N–H groups on the target loop. It is possible that these potential hydrogen bond interactions constitute the first stage of recognition, followed by the induced fit of the flexible linear peptide with the rigid aptamer ring. Since the spacing of the residues is regular and determined by the geometry of the peptide backbone for both the aptamer and its target, the degree of induced spatial adjustment required will be small. This type of accommodation will occur much more rapidly and reliably when at least one of the partners in the interaction is fairly rigid. The exact sequence of the peptide aptamer employed is shown in Figure 4 (structure A) and Table 1. The use of cysteines of different chiralities also encourages the ring to twist slightly, so that the H-bond acceptor carbonyl groups are turned away from the plane of the ring, making them available for binding to the linear peptide.

The sequence of this peptide aptamer was chosen with two purposes in mind. The first was to create a structure which could bind to other peptides via side chains using simple principles of complementarity, namely, preferential interactions occurring between side chains of opposite electrical polarity, or between side chains that are both aromatic, or both lipophilic. The second was to create a structure in which binding to its complementary peptide can be easily monitored, in this case, by including aromatic side-chain residues whose interaction with complementary residues can be measured by the observation of resulting changes in fluorescence, either of the peptide aptamer or of the ligand. Fluorescence of aromatic amino acid side chains is known to be sensitive to the environment; in the study conducted here, binding of the cyclic and linear peptides produced a marked change in the environment of both tyrosine and tryptophan due to their altered proximity to each other. See reference [10] for an overview of the use of fluorescence measurement techniques to study protein and peptide interactions. This structure is shown in Figure 4A, together with the sequence of a putative complementary linear peptide (Figure 4B). The basis for their binding relies on an exact match of the sequence order in both peptides, i.e., polar–lipophilic–aromatic–spacer–aromatic–lipophilic–polar. A serine spacer was included between the tryptophans to maintain the appropriate complementary spacing relative to the aptamer. Additional linear peptides were constructed in which the match with the cyclic peptide was imperfect, and binding interactions were compared as described in the following section. Proline was employed as the fourth amino acid to prevent the introduction of a side chain residue that could interfere with binding interactions between the complementary amino acids and to facilitate the formation of beta turns after cyclisation.

Figure 3 shows one of many ways in which the linear peptide can map onto the cyclic peptide, where spacing intervals allow both H-bonding interaction of the peptide links and interactions between relevant side-chain residues.

## 2. Results

Peptides that were synthesised are shown in Table 1. With the exception of structure A, all were linear peptides composed of natural amino acids in the L form.

Peptide B was designed to be complementary to cyclic peptide aptamer A, while peptides C to F have sequences that mismatch in some way with structure A. Peptide F was intended to be a nonbinding control, and peptides D and E were analogues of B and C, except that a proline was employed as a spacer group instead of a serine. It was anticipated that the spacer group in the linear peptide would align with the space above the lipidic side chain on the upper face of the cyclic peptide ring, where amino acid residues are absent. Finally, peptide G was the linear analogue of cyclic peptide A, with the same sequence of binding amino acid residues. All the linear peptides contained the same aromatic residues in the same positions in the chain to permit comparison of binding interactions across the whole series.

**Figure 5:**
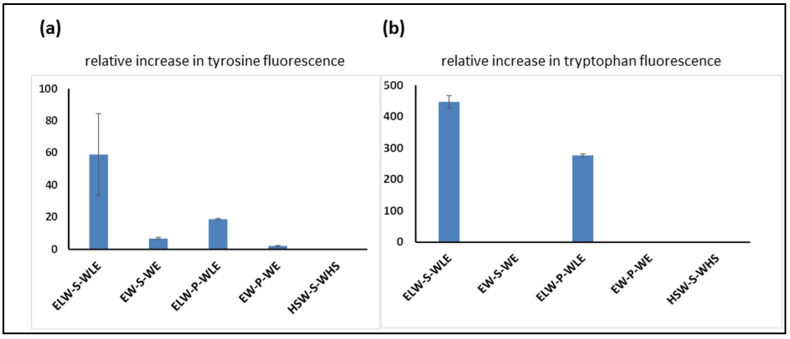
(**a**) Tyrosine fluorescence enhancement observed as a result of incubating cyclic peptide A at room temperature with the linear peptides shown. (**b**) Tryptophan fluorescence enhancement observed as a result of incubating cyclic peptide A at room temperature with the linear peptides shown.

In the first experiment, the peptide aptamer A was incubated with linear peptides B to F, and the tyrosine fluorescence enhancement was measured to compare the relative strengths of the binding interactions of each of the peptides to the cyclic peptide containing tyrosine. As was expected (Figure 5a), the strongest interaction was observed between peptide A and its fully complementary linear target, peptide B. Other linear peptides, in which complementary residues were missing or changed, showed a weaker interaction, and the replacement of serine by proline also reduced the level of interaction. This can be seen when comparing peptide B to peptide D, in which the binding residues are fully complementary, and the only difference is the nature of the central spacer amino acid. The same trends were seen when enhancement of tryptophan fluorescence was measured (Figure 5b).

**Figure 6:**
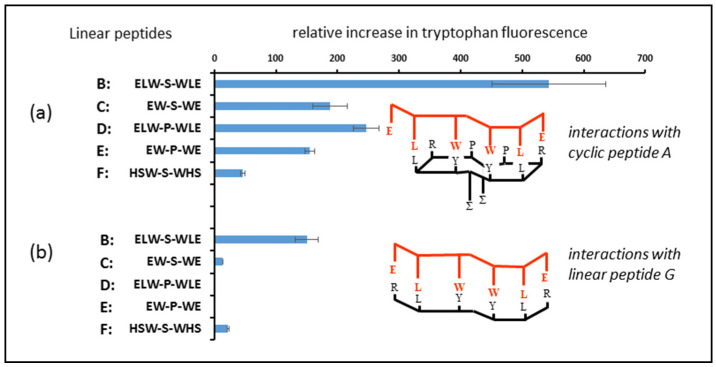
(**a**) Fluorescence enhancement observed as a result of incubating cyclic peptide A at 37 °C with the linear peptides shown. (**b**) Fluorescence enhancement observed as a result of incubating linear peptide G at 37 °C with the linear peptides shown. Tryptophan fluorescence is shown here, since tryptophan is the common feature across the whole series of linear peptides containing tryptophan, and these results are mirrored by the tyrosine readings obtained (data not shown).

In the second part of this experiment, an identical methodology was followed, except that peptides B to F were incubated with peptide aptamer A (Figure 6a) and peptide G (the linear analogue of peptide aptamer A). A higher incubation temperature was employed to ensure that the binding interactions reached equilibrium within a reasonable timeframe. This led to a slight discrepancy between the readings for the two experiments, but made no difference to the overall conclusions drawn. In all cases (Figure 6b), the relative binding of the peptides to the linear analogue of the cyclic peptide was much less than their interaction with the original cyclic peptide, demonstrating that the deployment of peptides in the cyclic form can result in stronger interactions with potential targets in biological systems.

## 3. Discussion and Conclusions

In our study, a change in fluorescence of tryptophan residues was employed to assess the relative strengths of the binding interactions between the peptides studied. Although this method does not permit the calculation of actual binding interaction energies, the magnitude of the differences observed is sufficient to prove the point, since these differences are marked. Other possible methods of evaluation (e.g., using fluorescence polarisation or equilibrium dialysis) are not practicable here because of the small sizes of the molecules involved.

Three basic observations were noted during this study. First, a cyclic peptide will bind more strongly to a linear amino acid chain than a linear peptide comprising the same sequence and spacing. Second, the strength of the interaction involved showed specificity in terms of the different sequences employed, demonstrating the validity of the approach adopted. Third, success or otherwise in binding is also sensitive to small changes in the target peptide (in this case serine to proline), which clearly influences the secondary structure of the peptide, despite the inherent flexibility of these peptides and their theoretical ability to conform to the structure to which they are binding.

Careful consideration was given to the sequence of the linear peptide chosen to act as the analogue of the cyclic peptide described in this study. As shown in Figure 1, the lipo-amino acids in the cyclic peptide do not participate in binding with the linear peptides, since they are sequestered within the cavity of the cyclodextrin ring. Consequently, lipo-amino acids were not included in the linear analogue. Even in the cyclic peptides, the inclusion of lipo-amino acids in the sequence renders them insoluble in aqueous phase, without recourse to cyclodextrin complexation, and a linear sequence containing all those amino acids included in the cyclic peptide would be highly insoluble on its own, since six out of the ten amino acid side chains would be extremely hydrophobic. Our previous attempts to convert such linear peptides into bioactive pseudo-cyclic peptides by adding cyclodextrin failed, and even if that approach had succeeded, their use would have defeated the object of the exercise here, since the aim was specifically to compare a rigid cyclic peptide with a flexible linear peptide, which is not in any constrained cyclic form. A linear peptide containing two lipo-amino acids would not only be totally insoluble in an aqueous phase, but it would also fold in on itself in a variety of random configurations, which would make the other amino acids (particularly the aromatics) unavailable for binding in any specific controlled fashion.

We also concluded, after careful modelling of how linear and cyclic peptides approach closely to each other, that the prolines also do not participate directly in binding to the linear peptides. The replacement of these prolines with glycines resulted in similar observations of the interaction profile of the cyclic peptide, which would not be the case if prolines significantly contributed to binding through the hydrophobicity of their side chains. We decided that the most appropriate linear analogue to employ was R-L-Y-S-Y-L-R, which provided the maximum opportunity to yield a soluble, flexible, and unconstrained chain in a random disordered configuration.

Since it was unclear whether optimal binding would occur when the chains were parallel (i.e., both in the N→C direction) or antiparallel, all the peptides employed (with the exception of the nonbinding construct F) were designed to be symmetrical about the central amino acid. We anticipated that a certain component of the binding interaction would be via hydrogen bonding between peptide linkages on complementary chains, and that this would help the linear peptide to locate the cyclic peptide and align itself appropriately to allow the side chain residues to interact. Hydrogen bonds are long compared to normal covalent bonds, and their influence might project a significant distance from the peptide chain via the intermediary of surrounding water molecules, provided that the potential bond-forming groups (C=O and N–H) are maintained in a fixed, immobile configuration, as they are in the cyclic peptide. See references [11,12] for demonstrations of long-range forces present in water in the range of 200–2000 nm. In contrast to electrostatic and hydrophobic interactions, hydrogen bonds are also highly directional, and presenting a peptide chain as a rigid scaffold may show that the potential hydrogen-bond-forming moieties are positioned in exactly the right places to initiate long-distance interactions, which can bring a linear peptide rapidly into the right orientation to test whether there is appropriate complementarity between the side chain residues.

Even when allowing for H-bonding interactions to take place, it appears that the presence of just two complementary side-chain residues is insufficient to achieve significant binding interaction on their own. This is shown by the lack of significant fluorescence enhancement seen when cyclic peptide A was incubated with peptide F, since, although the latter is described as a potentially nonbinding control, it still contains two tryptophans, which are potentially in the right place to bind with tyrosines on the cyclic peptide. The complementarity of four residues resulted in observable binding (Figure 5, peptide A with C), although not to the degree shown when all six residues matched.

The observations here may not hold for all cyclic peptides that can be constructed. Steps have been taken in this study to capitalise on the cyclic nature of the peptide to immobilise the peptide chain as much as possible, since we think that a rigid structure is important if it is to overcome the innate flexibility of linear peptides, which makes contending with thermal disruptions more difficult due to Brownian motion. Therefore, other cyclic peptides with larger rings, in which opportunities for cross-ring interactions such as H-bonding are lacking, may bind much less well to linear peptides than to the structures described here.

## 4. Materials and Methods

### 4.1. Reagents

All peptides were synthesised to >95% purity by Peptide & Protein Research Ltd., Farnborough U.K., and their identities were confirmed using mass spectrometry. Hydroxypropyl beta-cyclodextrin, transcutol, and thiourea were all purchased from Sigma UK Ltd. (Poole, U.K.).

### 4.2. Experimental Detail

The cyclic peptide A was complexed with cyclodextrin by dissolving β-hydroxpropyl-cyclodextrin in transcutol at a concentration of 80 mg/mL, and then adding 50 μL to 1 mg of the peptide and mixing to form a clear solution. We added 950 μL of 2 M thiourea (pH 7) to reach a final concentration of cyclic peptide of 1 mg/mL. An identical procedure was employed to prepare a solution of linear peptide G.

Linear peptides B to F were dissolved in distilled water at a concentration of 1 mg/mL. At this concentration, nonlinear concentration-dependent increases in fluorescence were not observed for individual peptides.

Incubation of the cyclic peptide with the linear peptides B to F was performed by dispensing 40 μL of cyclic peptide solution into wells of a black 96-well microplate, adding 120 μL of each linear peptide to these wells, and incubating for one hour either at room temperature or at 37 °C, as specified in the legends of the figures. Control solutions were prepared containing only one of each of the two components, and fluorescence enhancement was determined by subtracting the combined fluorescence of each of the separate solutions from that observed for the solution containing both components together. The results reported are the increase in fluorescence (arbitrary units) above the additive sum of the individual components. All solutions were prepared in triplicate.

Fluorescence measurements were made using a Spectromax Gemini fluorescence plate reader at 290 and 260 nm, the excitation wavelengths appropriate for tryptophan and tyrosine, respectively. Emissions were measured at the wavelengths of the peak maxima for the combination of the two compounds as a mixture: 360 nm for tryptophan and 350 nm when excited at the tyrosine wavelength. Tyrosine does not normally have an emission peak of 350 nm; the observation of this wavelength as a maximum suggests several factors, including off-peak excitation of tryptophan at 260 nm, and possible resonant energy transfer from tyrosine to tryptophan. An enhancement was seen for both tyrosine and tryptophan excitation wavelengths, and the same trends were shown over the series of linear peptides studied.

The CD spectrum of a diluted Lexicon cyclic peptide cyclo(sRC(C8)FsSrc(C8)fS) was acquired using an Applied Photophysics Chirascan Plus spectrometer (Leatherhead, U.K.). A 0.5 mm rectangular cell was employed in the region of 260–190 nm. The instrument was flushed continuously with pure evaporated nitrogen throughout the experiment. The following parameters were employed: a 1 nm spectral bandwidth, a 1 nm stepsize, and a 1.5 s instrument time-per-point. The CD spectrum was buffer-baseline subtracted and measured at 23 °C. The far-UV CD spectrum of the peptide was processed using Savitsky-Gorlay smoothing with a convolution width of 7 points.

## Figures and Tables

**Table 1:** Sequence structures of peptides used in this study.

A:	cyclo(RP-C[C8]-PRLY-c[C8]-YL)
B:	ELWSWLE
C:	EWSWE
D:	ELWPWLE
E:	EWPWE
F:	HSWSWHS
G:	RLYGYLR
